# The Volume-Regulated Anion Channel LRRC8 is Involved in the Initiation of Epidermal Differentiation and is Deregulated in Psoriasis

**DOI:** 10.1016/j.xjidi.2025.100357

**Published:** 2025-02-17

**Authors:** Magdalena Jahn, Victoria Lang, Oliver Rauh, Torsten Fauth, Claudia Buerger

**Affiliations:** 1 Department of Dermatology, Venerology and Allergology, University Hospital Frankfurt, Germany; 2 Plant Membrane Biophysics, Department of Biology, Technical University Darmstadt, Germany; 3 BRAIN Biotech AG, Zwingenberg, Germany; 4 Akribion Therapeutics GmbH, Zwingenberg, Germany

**Keywords:** Epidermal differentiation, Keratinocytes, Ion channel, LRRC8, SWELL1

## Abstract

Recent studies have shown that LRRC8A, the essential subunit of the volume-regulated anion channel LRRC8, which is responsible for mediating cell volume regulation during hypotonic stress, is predominantly localized in the basal layer of the epidermis. This prompted us to investigate whether LRRC8A plays a role in maintaining epidermal homeostasis by regulating key processes initiated in this layer, such as cell proliferation and/or differentiation.

LRRC8A was found to be strongly upregulated in transiently amplifying cells at the onset of differentiation. While *LRRC8A* mRNA remains high when keratinocytes mature further, the LRRC8A protein is drastically downregulated. Interference with *LRRC8A* expression at this step inhibits the transition of keratinocyte stem cells into transiently amplifying cells and impairs terminal differentiation. As psoriasis is a common chronic inflammatory skin disease characterized by disturbed epidermal differentiation and aberrant function of transiently amplifying cells, we investigated the involvement of LRRC8A in this disease. Indeed, LRRC8A was strongly decreased in lesional psoriatic skin, which could also be mimicked in vitro using Th1/Th17 cytokine mixes. Thus, our data suggest that LRRC8 could serve as a therapeutic target for the topical treatment strategies of psoriatic lesions by restoring the capacity of keratinocytes to initiate differentiation.

## Graphical abstract

**Figure:**
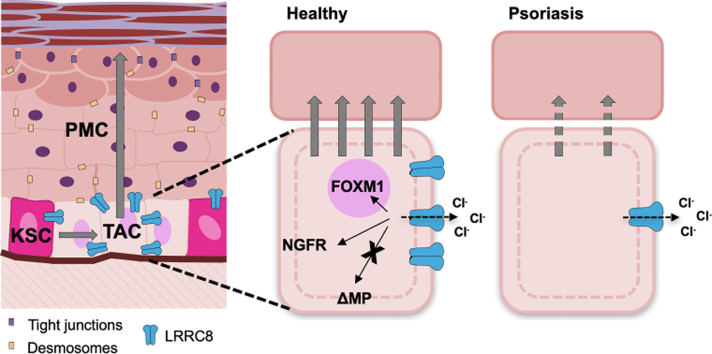

## Introduction

The human epidermis as the outermost barrier protects the body against external hazards such as pathogens, UV radiation, chemicals, or changes in temperature or humidity. To achieve this, the epidermis is constantly renewed by proliferating keratinocytes in the basal layer that differentiate into corneocytes eventually forming—together with lipids and moisturizing factors—a tight epidermal barrier (Moreci and Lechler, 2020). Within the basal layer, 2 subtypes of proliferative keratinocytes can be distinguished. Keratinocyte stem cells (KSCs) undergo asymmetrical cell division generating a daughter stem cell, which ensures lifelong regeneration of the epidermis and a transiently amplifying cell (TAC). This cell type proliferates for a limited period before leaving the stratum basale, committing to terminal differentiation and forming the epidermal barrier (Negri and Watt, 2022; Watt, 1998). Numerous signaling cascades have been described to influence keratinocyte proliferation and transition to differentiation; however, the underlying processes are not yet fully understood. Interestingly, transient amplifying cells (TACs) are enriched and display substantial molecular alterations in lesional psoriatic skin (PP) (Witte et al, 2020).

In addition to the physical barrier, skin cells possess further mechanisms to react to extrinsic stress factors. Changes in the osmotic balance can be compensated by regulating the activity of ion channels and thus adjusting the cell volume (Jahn et al, 2021). Upon exposure to hypotonic osmotic stress, volume-regulated anion channels (VRACs) are opened (Hoffmann et al, 2009), which allow the efflux of chloride and organic osmolytes, thereby changing the osmotic gradient, resulting in outflux of water and restoration of the original cell volume. This process is referred to as regulatory volume decrease (Jahn et al, 2021; Jentsch, 2016). About 10 years ago, leucine-rich repeat-containing protein 8 (LRRC8) was identified as a VRAC-forming protein complex, which is composed of up to 5 different subunits (LRRC8A–E) as a heteromeric multimer (Deneka et al, 2018; Voss et al, 2014), with LRRC8A being the essential subunit for ion channel function (Qiu et al, 2014; Voss et al, 2014). In addition to osmoregulation, LRRC8 is considered important in a plethora of cellular mechanisms in various cell types, like proliferation (Lu et al, 2019; Rubino et al, 2018), differentiation (Chen et al, 2019a; Kumar et al, 2014), migration (Zhang et al, 2018) and intracellular signaling pathways (Alghanem et al, 2021; Kumar et al, 2020; Zhang et al, 2017). However, ion channel composition and molecular functions vary a lot among cell types and tissues, thereby making it difficult to draw conclusions from one cell type to another (Lück et al, 2018; Pervaiz et al, 2019). It was recently described that LRRC8A is mainly localized in the basal layer of the human epidermis and contributes to cell volume regulation upon hypotonic stress in human keratinocytes (Trothe et al, 2018), which is well protected against environmental stress due to the upper, barrier forming layers. However, adjustments in cell volume can also occur without osmotic imbalances and are an integral part of many physiological processes, such as proliferation and differentiation (Hoffmann et al, 2009; Jentsch, 2016). Thus, we wondered whether the channel can potentially also be an important regulator of these processes in skin cells.

We found that LRRC8A is upregulated during the onset of differentiation and contributes to the transition from KSC to TAC. Consequently, silencing *LRRC8A* leads to reduced terminal differentiation. In lesional psoriatic skin, LRRC8A was markedly downregulated, which could be recapitulated in vitro, when keratinocytes were treated with Th1/Th17 cytokines. In summary, our data suggest that LRRC8A is a modulator of epidermal maturation and might represent an interesting anti-psoriatic target to restore proper differentiation.

## Results

### Upregulation of LRRC8A during differentiation initiation

**Figure 1:**
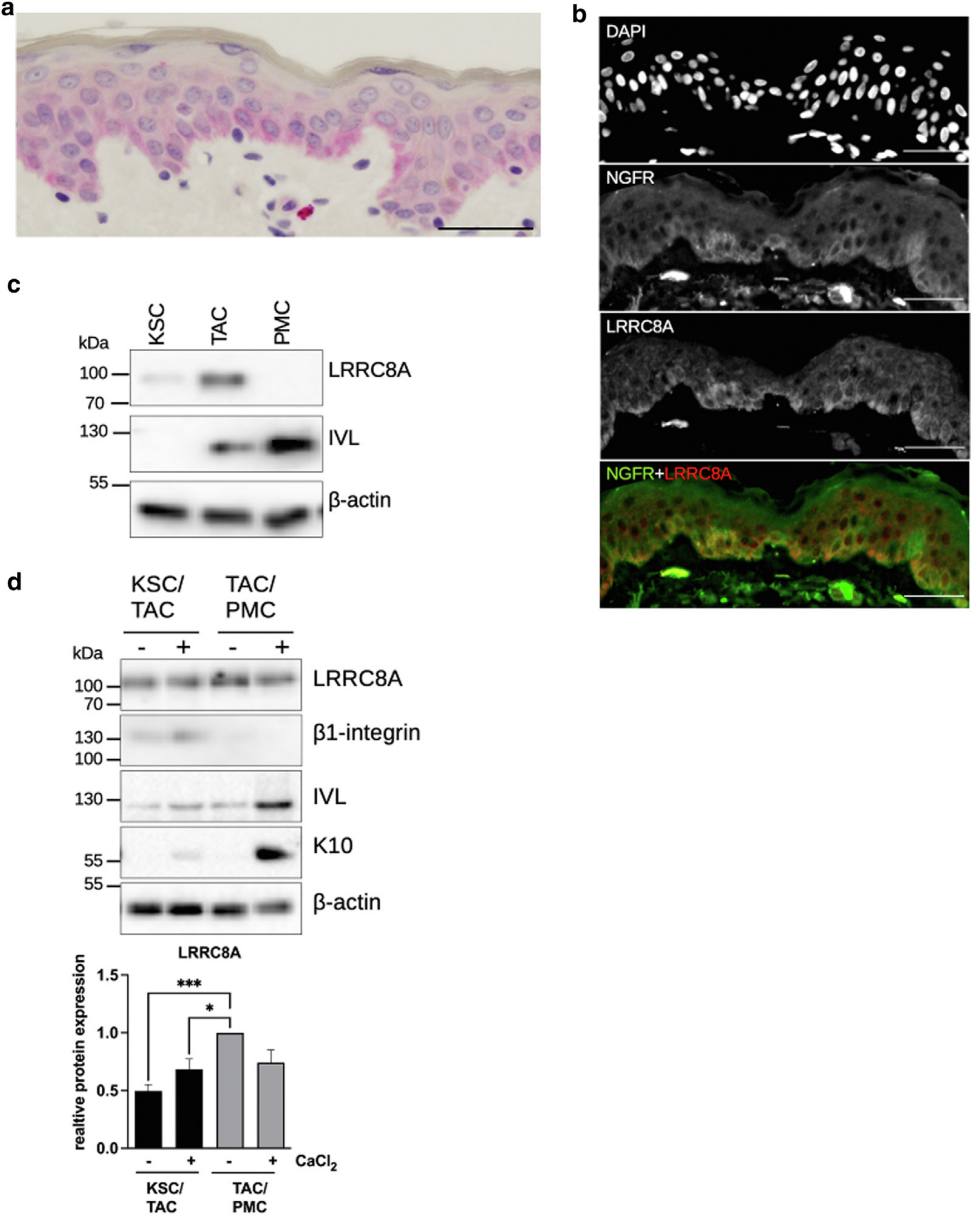
**LRRC8A protein is primarily localized in transient amplifying cells of the basal epidermal layer.** (**a**) LRRC8A was stained via immunohistochemistry in punch biopsies from healthy donors. LRRC8A is localized in the basal epidermal layer, as described previously (Jahn et al, 2021; Trothe et al, 2018). Bar represents 20 μm. (**b**) Native human skin was stained with a LRRC8A specific antibody (red) and an anti-CD271 antibody (green). Nuclei were stained with DAPI. Greyscale images are shown for each channel as well as an overlay image of the red and green channel. Scale bars represent 20 μm. (**c**) Keratinocyte populations were separated into KSC, TAC, and PMC based on their adhesion to collagen IV. Protein lysates from these populations were analyzed by Western blotting. LRRC8A was mainly detected in KSC and TAC, but not PMC, with the highest level in TAC. (**d**) Keratinocyte populations were differentiated by the addition of 2 mM CaCl_2_ for 72 hours, analyzed by Western blotting, and quantified by densitometry (n = 16 independent experiments, mean + SEM). KSC, keratinocyte stem cell; LRRC8A shows its highest expression in early (-CaCl_2_) TAC. LRRC8, leucine-rich repeat-containing protein 8; PMC, post-mitotic cell; TAC, transiently amplifying cell.

Building on previously published data, showing that LRRC8A is primarily localized in the basal epidermal layer (Jahn et al, 2021; Trothe et al, 2018) (Figure 1a), we investigated the expression of LRRC8A in this location in more detail. Primary keratinocytes (normal human keratinocytes [NHKs]) were separated into subpopulations based on their adhesion to type IV collagen and LRRC8A was measured in each population on the RNA and protein level. Consistent with its localization in situ (Figure 1a), we detected the LRRC8A protein only in the basal populations (KSC and TAC), but not in post-mitotic cells (PMCs), and found the highest expression in TAC (Figure 1c). This finding was further substantiated by the colocalization of LRRC8A with CD271/NGFR, a marker for TAC (Truzzi et al, 2011) in the basal layer of the human epidermis (Figure 1b). To model the maturation of basal keratinocytes from one stage to the next in vitro, Ca^2+^ was added to freshly isolated KSC and TAC respectively. Accordingly, we found that LRRC8A protein is specifically high during the transition from KSC to TAC and decreases, as soon as TAC starts to differentiate further (Figure 1d).

**Figure 2:**
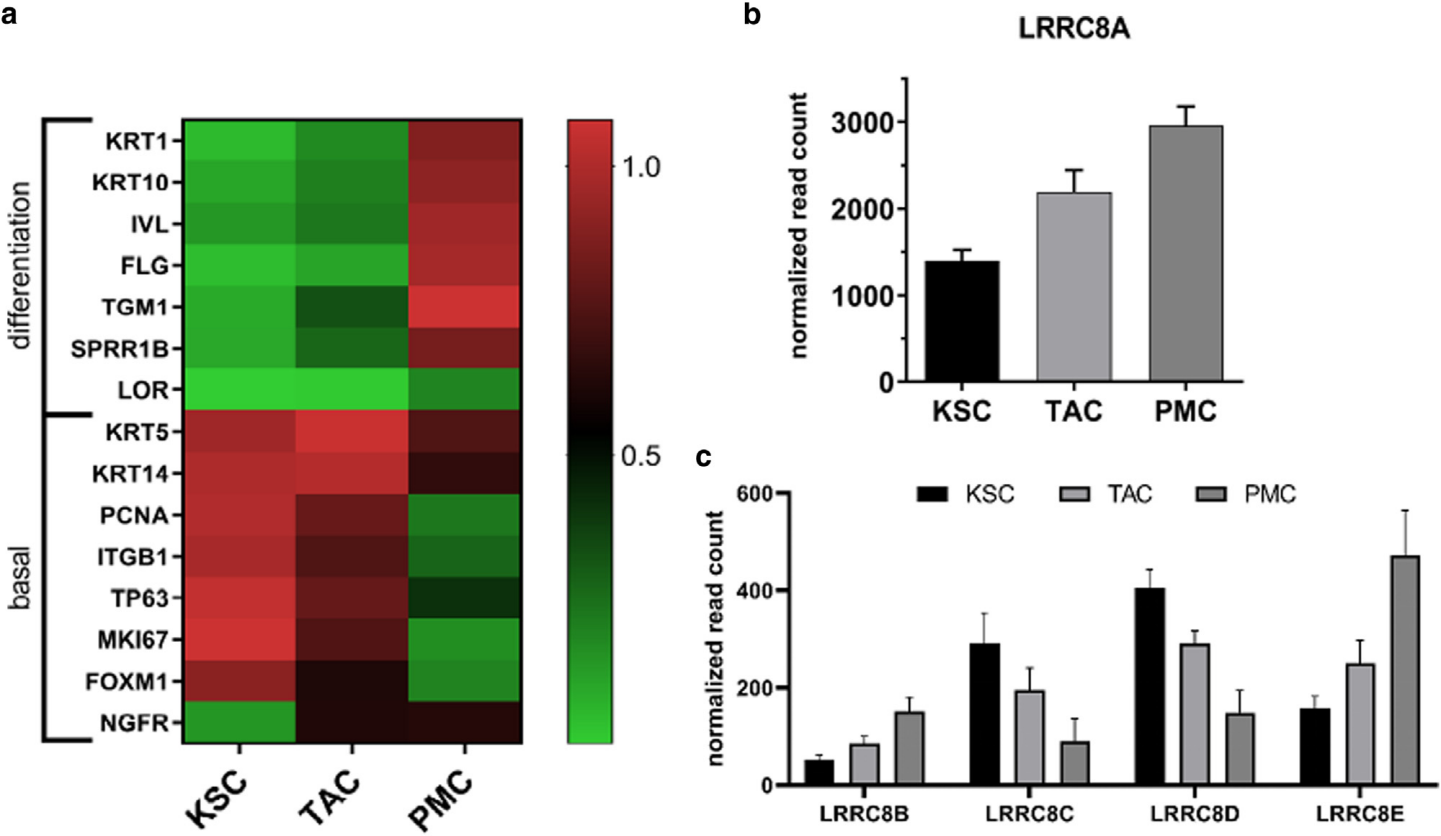
***LRRC8A* gene expression increases with progressing epidermal maturation.** RNA was isolated from keratinocyte populations and used for RNA sequencing (n = 4 independent experiments). Read counts were normalized and expression analysis was conducted using DESeq2. (**a**) To verify proper separation of keratinocyte populations, selected marker genes of basal or differentiating keratinocytes were normalized to the highest expressing population and compared to the others. Color changes from green to red indicate an increase in gene expression. (**b**) *LRRC8A* was differentially expressed among different populations (p: 3.6 × 10^−8^, Likelihood Ratio Test) with its transcripts significantly increasing during maturation. (**c**) All other LRRC8 family members, whose transcripts are severely less abundant than those of *LRRC8A*, are also differentially expressed among different populations (*P* values: B: 9.1 × 10^−9^, C: 5.8 × 10^−10^, D: 7.01 × 10^−8^, E: 1.31 × 10^−25^, Likelihood Ratio Test). *LRRC8B* and *LRRC8E* increase during differentiation, while *LRRC8C* and *LRRC8D* decrease. LRRC8, leucine-rich repeat-containing protein 8.

To investigate if this specific regulation can be also seen on the level of gene expression, each keratinocyte population was analyzed by RNA-sequencing and DESeq2 analysis (Figure 2). As expected, we found increased expression of differentiation markers such as involucrin (*IVL*), cytokeratin 1/10 (*KRT1* and *KRT10*), or filaggrin (*FLG*) and downregulation of markers of the basal layer such as basal keratins 5 and 14 (*KRT5* and *KRT14*), β1 integrin (*ITGB1*) or *PCNA*, during maturation from KSC to PMC. In addition, the KSC marker forkhead box protein M1 (*FOXM1*) was especially high in the KSC population, while the TAC marker *NGFR* increased when cells transitioned from KSC to TAC. Overall, this data indicates successful separation of the specific populations (Figure 2a). Transcription of LRRC8A increased from KSC to TAC, like the reported protein levels. Surprisingly, *LRRC8A* mRNA further augmented when TAC developed into PMC, suggesting that the downregulation of the LRRC8A protein in differentiating, PMCs occurs on the post-transcriptional level (Figure 2c). The other LRRC8 family members *LRRC8B*, *C*, *D*, and *E* showed overall lower transcript abundancies in all populations compared to *LRRC8A*, which is consistent with previous data (Jahn et al, 2021; Trothe et al, 2018). Like *LRRC8A*, both *LRRC8B* and *LRRC8E* displayed an increase in mRNA levels during keratinocyte maturation. In contrast, *LRRC8C* and *LRRC8D* decreased from KSC to PMC (Figure 2c).

**Figure 3:**
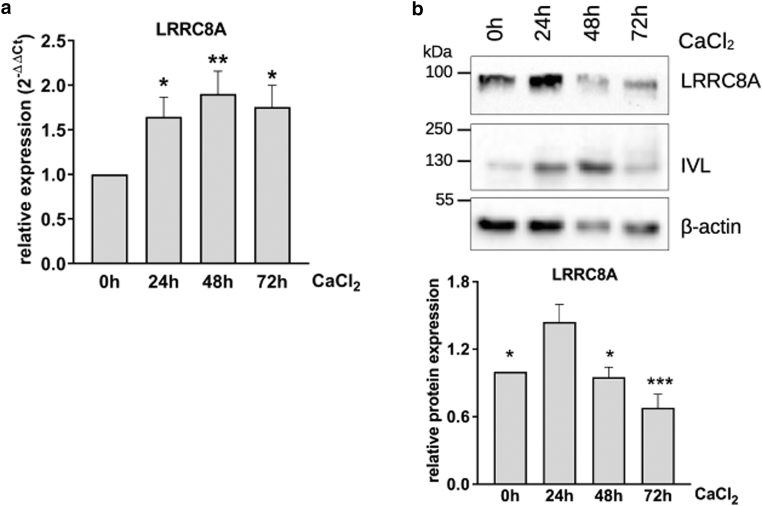
**LRRC8A gene and protein expression can be recapitulated in vitro.** Through the addition of 2 mM CaCl_2_, harvested every 24 hours, and analyzed via RT-qPCR, 5.7 × 10^4^/cm^2^ cells were differentiated (**a**) or Western blots (**b**). RNA is upregulated and stays increased during further differentiation (n = 11 independent experiments, mean + SEM) (Repeated measures ANOVA and Tukey post-test to correct for multiple comparisons, asterisks indicate changes compared to 0 hours [other comparisons n.s.]). In contrast, the amount of LRRC8A protein increases 24 hours after Ca^2+^ addition and is downregulated afterward. Blots were quantified by densitometry (b, bottom) (n = 7 independent experiments, mean + SEM) (Repeated measures ANOVA and Tukey post-test to correct for multiple comparisons, asterisks indicate changes compared to 24 hours [other comparisons n.s.]). LRRC8, leucine-rich repeat-containing protein 8; n.s., not significant.

To investigate whether the differentiation-dependent pattern of LRRC8A expression could be recapitulated with the entire keratinocyte population in vitro, NHK were differentiated by the addition of Ca^2+^ and LRRC8A was monitored at different time points (Figure 3). LRRC8A protein was strongly increased shortly after initiation of differentiation, as indicated by increased involucrin (IVL) expression. LRRC8A expression then rapidly decreased with further maturation, resulting in a bell-shaped expression pattern (Figure. 3b). Again, *LRRC8A* mRNA increased during the onset of differentiation but remained high with further maturation (Figure 3a) like the results obtained from PMC (Figure 2b).

In summary, we found a tight regulation of LRRC8A at the transitioning step from KSC to TAC, which made us hypothesize that LRRC8A might play a role during the switch from proliferation to differentiation in the basal epidermal layer.

### Disruption of LRRC8A expression impairs keratinocyte differentiation

**Figure 4:**
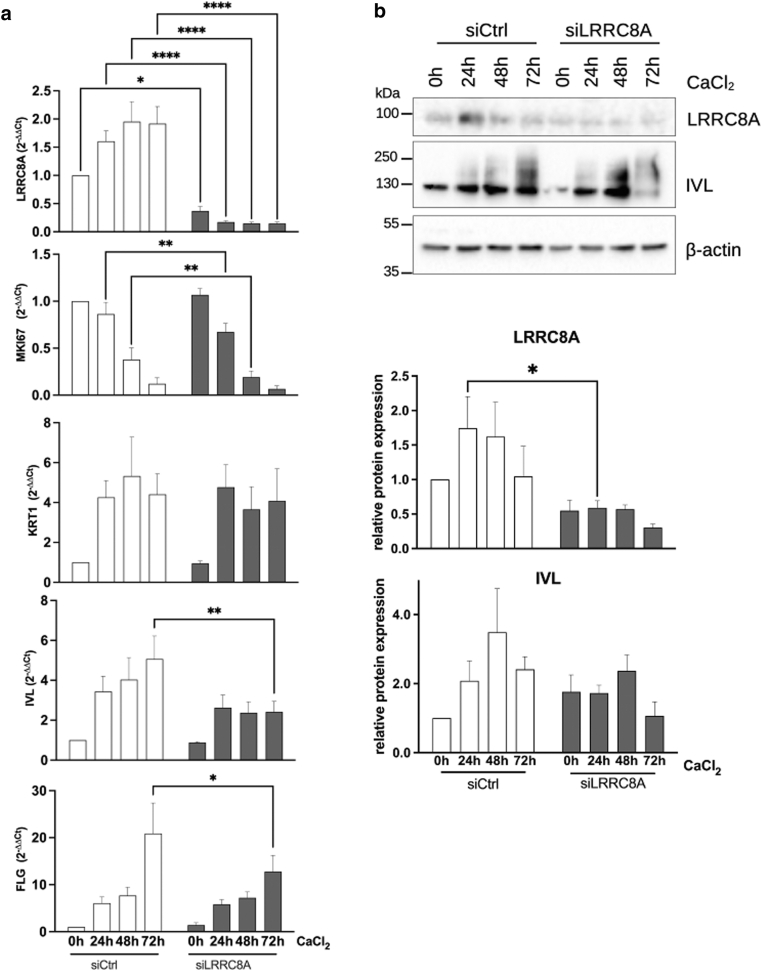
**Knockdown of *LRRC8A* interferes with epidermal differentiation.** Keratinocytes (5.7 × 10^4^/cm^2^) were seeded and transfected with *LRRC8A*-specific siRNA (siLRRC8A) or an unspecific siCtrl. Seven to 8 hours after transfection, cells were stimulated with 2 mM CaCl_2_ to induce differentiation, harvested every 24 hours, and analyzed by RT-qPCR (**a**) or Western blot (**b**). (**a**) Knockdown of *LRRC8A* reduces mRNA levels of *MKI67*, *IVL*, and *FLG*, with the strongest effect after 72 hours (n = 7–9 independent experiments, mean + SEM) (2-way ANOVA with Sidak post-test to correct for multiple comparisons). (**b**) Western blot analysis shows a slight reduction of IVL after 72 hours in knockdown cells. Blots were quantified by densitometry (n = 5–6 independent experiments, mean + SEM) (2-way ANOVA with Sidak post-test to correct for multiple comparisons). *FLG*, filaggrin; *IVL*, involucrin; LRRC8, leucine-rich repeat-containing protein 8; *MKI67*, marker of proliferation Ki-67; siCtrl, siRNA control; siLRRC8A, *LRRC8A-*specific siRNA; siRNA, small interfering RNA.

**Figure 5:**
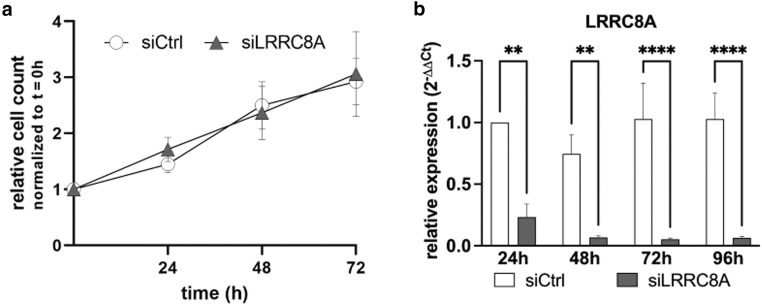
**Knockdown of *LRRC8A* does not interfere with keratinocyte proliferation.** Primary keratinocytes (2.1 × 10^4^/cm^2^) were seeded and transfected 24 hours later with 10 pmol siRNA specific for *LRRC8A* (siLRRC8A) or unspecific siCtrl. (**a**) Cells were trypsinized and counted every 24 hours. Cell count was normalized to t = 0 hours (first harvest 8 hours after transfection). No differences between siCtrl and siLRRC8A were detected (n = 7 independent experiments, mean ± SEM). (**b**) After counting cell numbers for proliferation, RNA was isolated and knockdown was verified via RT-qPCR (n = 7 independent experiments, mean + SEM) (2-way ANOVA with Sidak post-test to correct for multiple comparisons). LRRC8, leucine-rich repeat-containing protein 8; siCtrl, siRNA control; siLRRC8A, *LRRC8A-*specific siRNA; siRNA, small interfering RNA.

To assess whether this remarkable LRRC8A expression pattern contributes to the tightly controlled switch from proliferation to differentiation, we used small interfering RNA (siRNA)–mediated knockdown of *LRRC8A* and analyzed the ability of these keratinocytes to proliferate or commit to keratinocyte maturation using siRNA mediated *LRRC8A* downregulation. Since differentiation begins 12 to 24 hours after Ca^2+^ stimulation (Buerger et al, 2017) and *LRRC8A* expression is upregulated during this period, we inhibited its expression before this increase. Thus, NHK were transfected with *LRRC8A*-specific siRNA (siLRRC8A) 8 hours before stimulation, with successful knockdown being confirmed at both the RNA and protein levels (Figure 4a and b). siRNA-mediated reduction of *LRRC8A* led to the reduction of the proliferation marker *MKI67* mRNA (Figure 4a). Thus, the growth of siRNA-transfected cells was monitored by counting cell numbers over time followed by the calculation of doubling times from the generated growth curves. However, no difference between control (siCtrl) and knockdown cells (siCtrl 41.5 ± 5.3 hours and siLRRC8A 42.4 ± 9.1 hours; mean ± SEM, n = 5 independent experiments) (Figure 5a) could be detected. We conclude that LRRC8A is not crucially involved in regulating proliferation in the basal layer.

**Figure 6:**
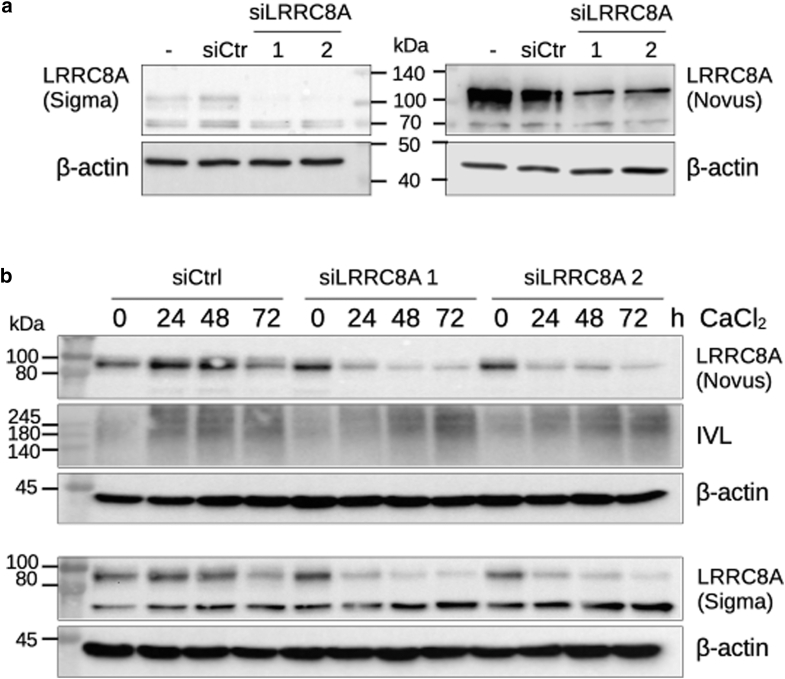
**Efficient knockdown of *LRRC8A* with 2 specific siRNAs interferes with the expression of differentiation markers.** (**a**) Keratinocytes (5.7 × 10^4^/cm^2^) were seeded and transfected with 2 *LRRC8A* siRNAs (siLRRC8A 1 and 2) or an unspecific siCtrl or left untreated and harvested after 48 hours. Protein lysates were subjected to Western blotting with the indicated antibodies. (**b**) Keratinocytes (5.7 × 10^4^/cm^2^) were seeded and transfected with 2 *LRRC8A* siRNAs or an unspecific siCtrl. Seven to 8 hours after transfection, cells were stimulated with 2 mM CaCl_2_ to induce differentiation, harvested every 24 hours, and analyzed by Western blotting with the indicated antibodies. LRRC8, leucine-rich repeat-containing protein 8; siCtrl, siRNA control; siLRRC8A, *LRRC8A-*specific siRNA; siRNA, small interfering RNA.

Next, we looked at the impact of LRRC8A on differentiation. While the expression of *KRT1* was not affected (Figure 4a), knockdown of *LRRC8A* led to reduced expression of the late differentiation markers such as *IVL* and *FLG* (Figure 4a), with the effect being strongest after 72 hours of differentiation. However, the decrease of *IVL* was more pronounced at the RNA level, while the decrease of the protein was weaker (statistically not significant) (Figure 4b) but could be additionally confirmed with a second siRNA that showed a similar trend on the IVL protein (Figure 6b).

This data suggests that, although LRRC8A does not affect keratinocyte proliferation, it does contribute, at least in part, to epidermal differentiation. Since LRRC8A is upregulated during the very early onset of differentiation but impacts later differentiation events such as IVL and filaggrin expression, we hypothesize that LRRC8A does not directly regulate IVL/filaggrin itself but modulates other early differentiation events that eventually lead to a decrease in differentiation.

### LRRC8A contributes to the transition from stem cells to transiently amplifying cells

To investigate this hypothesis, we additionally analyzed early regulators of epidermal differentiation. These include NGFR (also known as CD271 and p75NTR), an NGF (neuronal growth factor) receptor that is specifically upregulated in early TAC (Lotti et al, 2022; Truzzi et al, 2015) and reduced in lesional PP (Truzzi et al, 2011), and the recently identified forkhead box protein M1 (Enzo et al, 2021; Lotti et al, 2022), an oncogenic transcription factor (Kalathil et al, 2020) that contributes to proliferative capacity (Smirnov et al, 2016).

**Figure 7:**
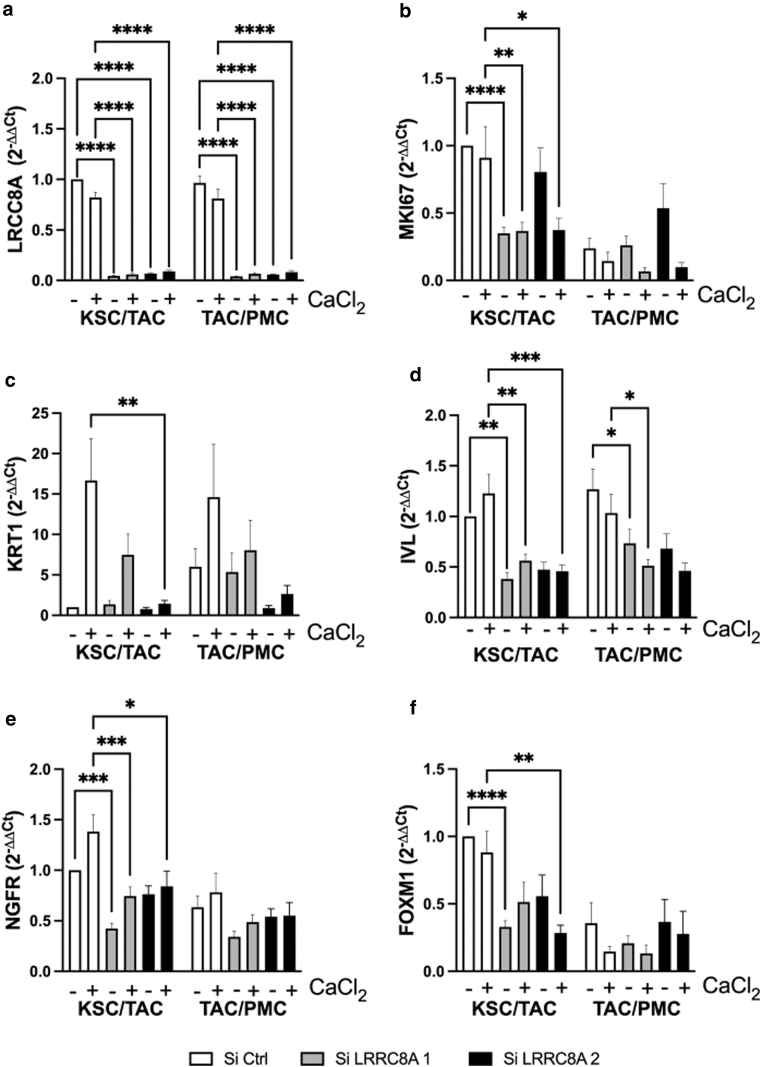
**LRRC8A modulates the transition from KSC to TAC.** Keratinocyte populations were separated based on their adhesion to collagen IV, transfected with 2 different *LRRC8A* specific siRNAs (siLRRC8A 1 and 2) or an unspecific siCtrl, and differentiated with 2 mM CaCl_2_. RNA was isolated after 72 hours and analyzed by RT-qPCR for the indicated genes. Knockdown of *LRRC8A* (**a**) decreases expression of *KRT1* (**c**) and *IVL* (**d**) in all populations, although it was not significant in all cases. Markers of basal cell types such as *MKI67* (**b**), *NGFR* (**e**), and *FOXM1* (**f**) were reduced in *LRRC8A* knockdown with both siRNAs in cells transitioning from KSC to TAC (KSC/TAC) but not in cells transitioning from TAC to PMC (TAC/PMC) indicative of a reduced entry into the differentiation process (n = 7 to 17 independent experiments, mean + SEM) (2-way ANOVA with Tukey post-test to correct for multiple comparisons). *FOXM1*, forkhead box protein M1; *IVL*, involucrin; *KRT1*, cytokeratin 1; KSC, keratinocyte stem cell; LRRC8, leucine-rich repeat-containing protein 8; *MKI67*, marker of proliferation Ki-67; *NGFR*, neuronal growth factor receptor; PMC, post-mitotic cell; siCtrl, siRNA control; siLRRC8A, *LRRC8A-*specific siRNA; siRNA, small interfering RNA; TAC, transiently amplifying cell.

Since LRRC8A is mainly upregulated during the transition from KSC to TAC, we specifically wanted to interfere at this stage. Therefore, isolated KSC and TAC were transfected with 2 *LRRC8A*-specific siRNAs, and differentiation was initiated by adding CaCl_2_ whereby the cells developed from KSC into TAC or from TAC into PMC, respectively. Both siRNAs mediated comparable reduction of *LRRC8A* mRNA (Figure 7a) and protein (Figure 6a and b). RT-qPCR analysis showed that *LRRC8A*knockdown again downregulated the proliferation marker *MKI67* mRNA (Figure 7b), which was shown to be especially high in TAC compared to KSC (Lotti et al, 2022). Supporting our previous results, siRNA-transfected cells exhibited reduced expression of *KRT1* and *IVL* (Figure 7c and d). Interestingly, in the absence of *LRRC8A*, *NGFR*, and *FOXM1* were strongly downregulated (Figure 7e and f). In particular, silencing hindered the upregulation of these genes during the transition from KSC to TAC, while the subsequent downregulation during the transition from TAC to PM cells was not further reduced (Figure 7e and f). These data clearly indicate that LRRC8A is involved in an early stage of keratinocyte differentiation.

### Regulation of LRRC8A in the human epidermis is disturbed in TH1 inflammation and psoriatic skin

**Figure 8:**
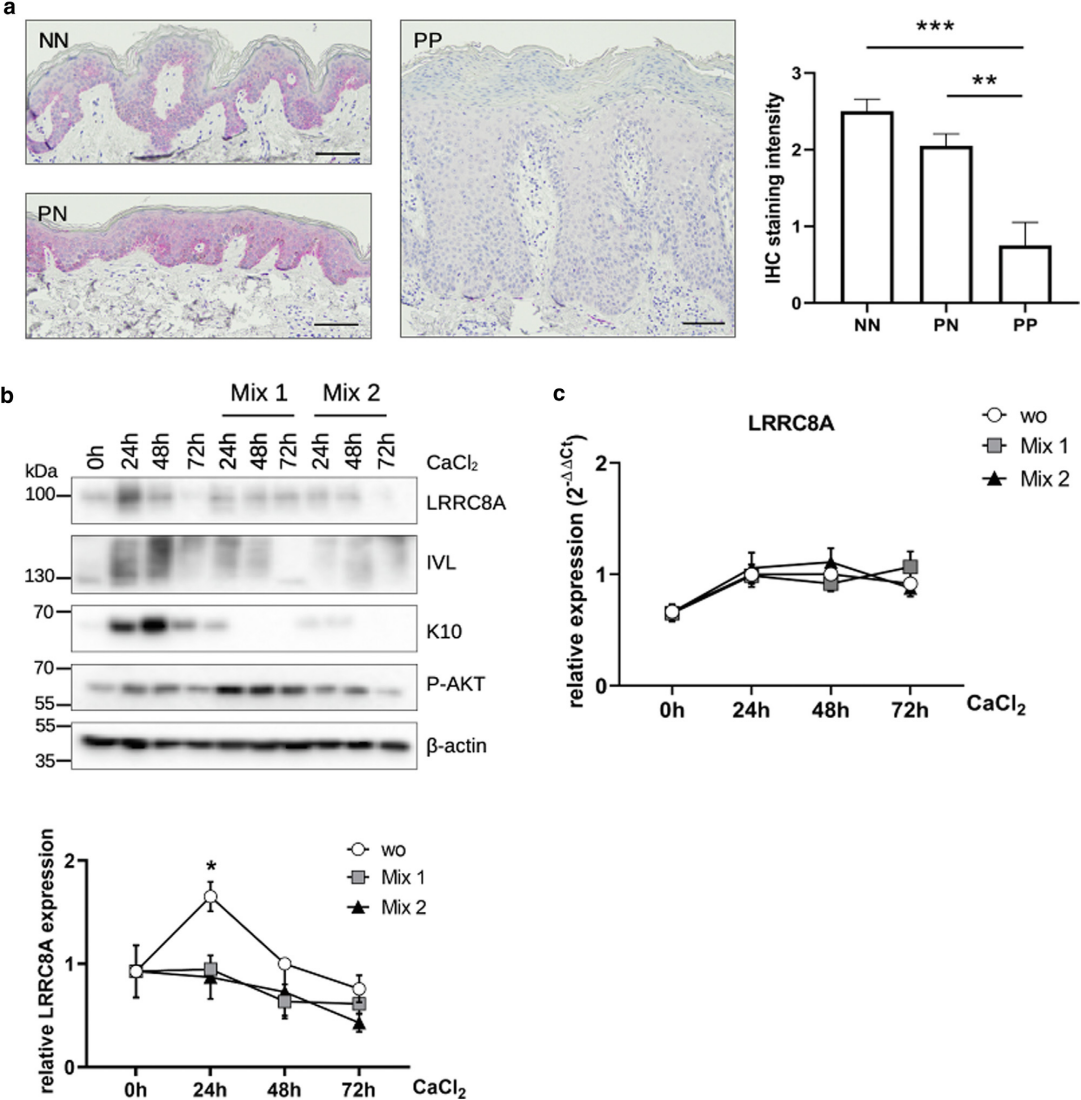
**LRRC8A regulation during epidermal differentiation is disturbed in an inflammatory environment.** (**a**) LRRC8A was stained in punch biopsies from NN and PP or PN skin from psoriasis vulgaris patients using anti-LRRC8A antibody. Representative staining was selected (left). Scale bars represent 50 μm. Staining intensities were estimated semi-quantitatively on a scale from 0 to 3 (right) (n = 5 to 10 independent experiments, mean + SEM) (1-way ANOVA with Tukey post-test to correct for multiple comparisons). (**b, c**) In KGM-2, 5.7 × 10^4^/cm^2^ cells were differentiated by Ca^2+^ without supplements in the presence or absence of Th1 cytokines (Mix 1 and 2) for the indicated time points. Mix 1 consisted of IL-1β, IL-17A, and TNF-α, and Mix 2 of IL-22 and TNF-α (20 ng/μl each). (**b**) Protein lysates were subjected to Western blotting with antibodies for K10 and IVL, to show impaired differentiation and AKT activation under inflammatory conditions. Densitometric quantification of blots indicates that LRRC8A upregulation was inhibited by cytokines (n = 6 to 8 independent experiments, mean + SEM) (2-way ANOVA with Sidak post-test to correct for multiple comparisons). (**c**) RT-qPCR in contrast showed that *LRRC8A* mRNA levels are not dysregulated by Th1 inflammation (n = 4 to 6 independent experiments, mean ± SEM). AKT, serine/threonine proteine kinase homolog to v-akt1 oncogene; LRRC8, leucine-rich repeat-containing protein 8; NN, healthy donors; PN, non-lesional skin; PP, lesional skin.

In the psoriatic lesions, TAC are strongly expanded and show an altered molecular signature, which seems to be responsible for the epidermal defects in psoriasis such as enhanced basal proliferation and aberrant keratinocyte differentiation in suprabasal layers (Franssen et al, 2005; Grabe and Neuber, 2007; Witte et al, 2020). Since we provide evidence for the role of LRRC8A in this cell type, we wondered whether LRRC8A is deregulated in the skin of patients with psoriasis vulgaris. Strikingly, immunohistochemical staining of lesional PP indeed showed a strong decrease of LRRC8A expression, whereas the uninvolved skin of patients with psoriasis showed basal localization of LRRC8A and less expression in upper layers, like in healthy epidermis (Figure 8a). Since psoriasis is mediated by aberrant expression of Th1 cytokines, we investigated whether these inflammatory mediators also interfere with *LRRC8A* expression. NHKs were differentiated in the presence of different cytokine mixes consisting of IL-1β, IL-17A, and TNF-α (Mix 1) or IL-22, and TNF-α (Mix 2), which both abolished upregulation of LRRC8A at the onset of keratinocyte differentiation (Figure 8b). This regulation, however, cannot be detected when measuring RNA (Figure 8c), indicating again, that post-transcriptional regulation is of major importance for LRRC8A modulation in keratinocytes.

### LRRC8A-mediated ion flow does not play a role during epidermal differentiation

**Figure 9:**
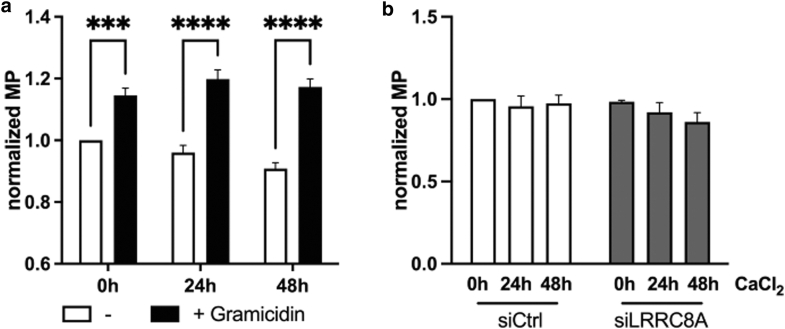
**The membrane potential does not change during keratinocyte differentiation.** (**a**) A sample of 1.5 × 10^4^ was differentiated with 2 mM CaCl_2_ 24 hours after seeding in 96-well plates. At the indicated time points cells were stained for 60 minutes with 1 μM DiBAC_4_(3), and fluorescence was recorded as a measure for the MP. Afterwards, 20 μg/ml gramicidin was added for 30 minutes and the measurement was repeated. Fluorescence values were normalized to untreated cells (0 hours) (n = 7 independent experiments, mean ± SEM). (**b**) A sample of 1.5 × 10^4^ keratinocytes was seeded in 96-well plates and transfected with siRNA after attachment overnight and treated with 2 mM CaCl_2_. Cells were stained for 60 minutes with 1 μM DiBAC_4_(3), fluorescence measured, and normalized to siCtrl cells without Ca^2+^ (0 hours) (n = 5 independent experiments, mean ± SEM). MP, membrane potential; siCtrl, siRNA control; siLRRC8A, *LRRC8A-*specific siRNA; siRNA, small interfering RNA.

Next, we aimed at elucidating how LRRC8A mediates its effects on TAC function. In myoblasts, it was shown, that LRRC8 activity promotes shifts in membrane potential due to ion shuttling, which, in turn, is a prerequisite for proper maturation (Chen et al, 2019a). To our knowledge, there is no data about changes in membrane potential during keratinocyte maturation. Thus, we examined whether LRRC8 ion channel activity, namely Cl^−^ flow over the cell membrane impacts keratinocyte membrane potential, which then might regulate keratinocyte differentiation. First, we measured membrane potential changes during the first 48 hours of differentiation using DiBAC_4_(3), a voltage-sensitive fluorescent dye. However, we did not find any changes in the membrane potential during the onset of differentiation (Figure 9a). To validate these measurements, the ionophore gramicidin was added after each time point, and fluorescence was measured again (Klapperstück et al, 2009). Gramicidin treatment results in an increase in relative membrane potential, indicating depolarization. Therefore, the depicted values represent a measure of the cell membrane potential (Figure 9a).

When *LRRC8A* was silenced, the membrane potential decreased slightly, but not statistically significant (Figure 9b). Overall, this data indicates that LRRC8A may contribute to stabilizing the membrane potential in keratinocytes, but membrane potential changes do not seem to be a relevant mechanism during epidermal differentiation.

In summary, LRRC8A contributes to the early onset of keratinocyte differentiation by regulating critical mechanisms during the transition from KSC to TAC as downregulation of LRRC8A in these cells blocks further progression and eventually results in aberrant differentiation. Interestingly, in lesional PP, LRRC8A protein levels are also strongly reduced, which seems to be mediated by Th1/Th17-cytokines. Therefore, we hypothesize that aberrant LRRC8A regulation might contribute to the pathogenesis of psoriasis, and hence LRRC8A might represent a promising target for the treatment of PP.

## Discussion

We show here for the first time that LRRC8A is upregulated when basal epidermal cells commit to differentiation. Interestingly, divergent levels of *LRRC8A* RNA and protein were found. While mRNA levels increased with progressing epidermal maturation, the LRRC8A protein is downregulated when cells leave the proliferative, basal compartment. This suggests a post-transcriptional mechanism regulating the LRRC8A channel protein for example via protein stability. Interestingly, regulation of ion channel activity by proteolysis has already been demonstrated for several ion channels, such as the voltage-regulate Ca^2+^ channel TRPC5 and Cav1.2 (Wang and Yule, 2018). This is potentially mediated via a similar mechanism that was found for viral proteins being able to target VRAC subunits for proteasomal degradation (Blest et al, 2024).

The upper, barrier-forming keratinocyte layers protect the stratum basale from environmental hazards and basal epidermal cells probably experience hypotonic stress only rarely, probably only after injury (Jahn et al, 2021). However, adjustments in cell volume can also occur without osmotic imbalances and are an integral part of many physiological processes, such as proliferation and differentiation (Hoffmann et al, 2009; Jentsch, 2016). Regarding LRRC8A’s involvement in proliferation, different results have been described depending on the analyzed cell type. While LRRC8A was shown to influence proliferation in certain cancerous diseases (Lu et al, 2019; Rubino et al, 2018), it was found to be dispensable in other cell lines (Liu and Stauber, 2019). In this study, we found that *LRRC8A* knockdown interferes with *MKI67* expression, which, however, is not sufficient to interfere with the overall proliferation of primary keratinocytes.

In contrast to its variable role in proliferation, growing evidence suggests that LRRC8 plays a key role in cell differentiation across several cell types (Chen et al, 2019b). Before its identification as an essential subunit of VRACs (Qiu et al, 2014; Voss et al, 2014), LRRC8A was actually attributed to B- and T-cell maturation (Kumar et al, 2014; Sawada et al, 2003), while LRRC8C was associated with adipocyte differentiation (Tominaga et al, 2004). Since 2014, LRRC8A has been further found to be involved in skeletal muscle cell differentiation (Chen et al, 2020, 2019a; Kumar et al, 2020) and late spermatogenesis (Lück et al, 2018). Our data show that LRRC8A is relevant during the early differentiation of keratinocytes. Initial evidence suggests reduced expression of LRRC8A in PP, where TACs are also altered (Witte et al, 2020). We hypothesize that upregulation of LRRC8A is crucial for the transition of KSCs to TACs and their further maturation, with reduced expression under inflammatory conditions potentially contributing to the pathogenesis of inflammatory dermatoses.

There is limited evidence of how signals are transmitted from membrane-bound ion channels to the nucleus to regulate genes in the epidermal differentiation complex and influence keratinocyte maturation. One possibility is that ion channel activity itself plays a role as seen in myoblast differentiation, where LRRC8 ion channel activity contributes to membrane potential shifts leading to hyperpolarization necessary for differentiation (Chen et al, 2020, 2019a). Keratinocytes which are non-excitable cells with a membrane potential of around −30 mV (Gönczi et al, 2007) undergo membrane hyperpolarization under hypotonic stress, which promotes proliferation but suppresses differentiation (Gönczi et al, 2007). However, changes in membrane potential during physiological differentiation have never been investigated to our knowledge. Our results, using the fluorescent membrane potential indicator DiBAC_4_(3) showed no significant changes in membrane potential during the first 48 hours of differentiation, suggesting that LRRC8A likely does not act through this mechanism.

We rather hypothesize that the intracellular LRR domain of LRRC8A interacts with downstream signaling proteins to transmit signals toward the epidermal differentiation complex. In several cell types, LRRC8A modulates PI3K/AKT signaling by interacting with the adaptor molecule GRB2 through its LRR domain (Alghanem et al, 2021; Kumar et al, 2020, 2014; Zhang et al, 2017). Since PI3K/AKT signaling is involved in keratinocyte differentiation (Calautti et al, 2005) and is hyperactivated in various inflammatory skin diseases like psoriasis (Buerger et al, 2012; Roy et al, 2023), it may link LRRC8A to epidermal differentiation. Given that LRRC8 ion channel activity is allosterically modulated by the LRR domain (Deneka et al, 2021) and potentially involves conformational changes (Gaitán-Peñas et al, 2016; König et al, 2019), ion channel function and intracellular signaling may influence each other.

We showed that LRRC8A is reduced in PP lesions. Interestingly, psoriatic lesions frequently occur at mechanically stressed body sites such as knees or elbows and there is increasing evidence that impaired mechanotransduction contributes to the pathogenesis of inflammatory dermatoses (Shutova and Boehncke, 2022). LRRC8A is induced in HEK293 cells under mechanical stress, such as hydrostatic pressure, and may mediate mechanical signaling (Park et al, 2022). In some cell types, LRRC8A interacts with caveolin-1 (Alghanem et al, 2021; Rezola et al, 2021; Zhang et al, 2017), a protein in caveolae that serves as a signaling platform for mechanoprotection (Parton et al, 2018). In addition, VRACs appear to be associated with β1-integrins, which also respond to mechanical stretch in different cell types (Browe and Baumgarten, 2003; Häussinger et al, 2006; Neveux et al, 2010; Sørensen et al, 2015). Thus, changes in cellular architecture during the transition from KSCs to TACs could be mechanical signals that activate LRRC8A. Therefore, reduced expression of LRRC8A in the context of psoriatic inflammation might amplify the effects of mechanical stress and thus contribute to the exacerbation of psoriatic plaques at these body sites.

A *LRRC8A*-knockout mouse model shows epidermal hyperkeratosis (Kumar et al, 2014), indicating severe defects in the absence of *LRRC8A* in vivo. In contrast, the defects observed in our in vitro *LRRC8A* siRNA knockdown were more subtle, likely because the knockdown did not fully eliminate the protein. In addition, our experiments were devoid of the above-mentioned stimuli such as movement, stretching, bending, or pressure, to which skin is constantly exposed in vivo.

Our findings suggest that activating LRRC8 ion channels could aid keratinocyte maturation and counteract the severe downregulation of LRRC8A in psoriatic lesions. Zinc pyrithione, recently identified as an LRRC8A activator (Figueroa and Denton, 2021), has shown benefits in mild psoriasis (Sadeghian et al, 2011). We therefore tested Zinc pyrithione’s effect on keratinocyte maturation. However, the effective Zinc pyrithione concentration for LRRC8 activation was cytotoxic to keratinocytes in both 2-dimensional and 3-dimensional assays (data not shown). Similarly, the VRAC inhibitor DCPIB (4-[2-butyl-6,7-dichlor-2-cyclopentyl-indan-1-on-5-yl]oxybutyric acid) also proved to be toxic (Decher et al, 2001). Nevertheless, it might be beneficial to screen for substances that are well-tolerated and capable of enhancing LRRC8A activity and therefore might work as topical anti-psoriatic ointments.

**Figure 10:**
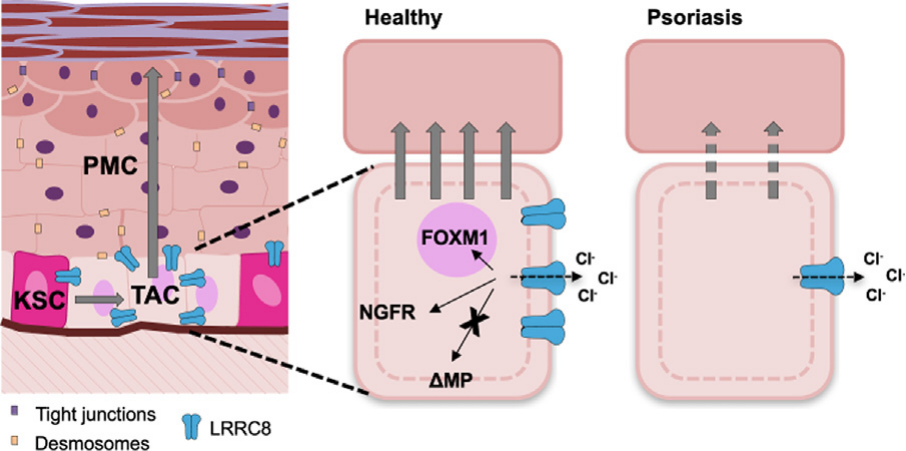
**LRRC8A is involved in early differentiation.** LRRC8A is upregulated when KSCs divide and develop into TACs, where LRRC8A interacts with essential factors of early differentiation such as FOXM1 and NGFR. We hypothesize that this is mediated by the interaction of downstream signaling molecules and not by the ion channel function of LRRC8A itself, because the MP is not altered by the absence of LRRC8A. In psoriatic skin, LRRC8A is less abundantly expressed, which potentially contributes to the differentiation defects seen in these skin lesions. FOXM1, forkhead box protein M1; KSC, keratinocyte stem cell; LRRC8, leucine-rich repeat-containing protein 8; MP, membrane potential; NGFR, neuronal growth factor receptor; PMC, post-mitotic cell; TAC, transient amplifying cell.

In summary, we found that the VRAC LRRC8 plays a role in epidermal maturation. The key subunit LRRC8A is most highly expressed during the transition from KSCs to the TAC population. Reduced LRRC8A interferes with this early differentiation process resulting in aberrant terminal differentiation. Notably, Th1 inflammation decreases LRRC8A, suggesting it could be a potential target for anti-psoriatic therapy (Figure 10). Future research will explore the molecular mechanisms linking LRRC8A to differentiation and whether ion channel activity itself and/or protein interaction via the LRR domain are critical for proper keratinocyte maturation.

## Materials and Methods

### Antibodies

LRRC8A antibodies were obtained from Novus Biologicals (NBP2-32158) or Sigma Aldrich (HPA016811—discontinued). The specificity of both antibodies was validated by knocking down LRRC8A using 2 siRNAs, which resulted in a specific reduction of a band at the expected size (Figure 6). IVL (ab20202) and keratin 10 (ab76318) antibodies were obtained from Abcam. β1-integrin (9699) and phospho-AKT (4060) antibodies were from Cell Signaling Technology. β-actin antibody (A1978) was from Sigma Aldrich. CD271/NGFR antibody was purchased from Thermo Fisher Scientific (MA5-13314).

### Cells and cell culture

Primary NHK (normal human keratinocytes) were isolated from the human juvenile foreskin or various adult body regions after skin reduction surgery. Cells were cultivated in KGM-2 (PromoCell).

Keratinocyte populations were separated based on their ability to attach to basal lamina proteins (Tiberio et al, 2002). Culture plates were coated with 100 μg/ml collagen IV from the human placenta (dilution from 1 mg/ml in 0.5 M acetic acid) in DPBS for 2 hours at 37 °C or at least 24 hours at 4 °C. Cells attaching within 5 minutes at 37 °C were considered KSC. Non-attached cells were transferred into another coated well, cells attached overnight were regarded as TACs, while cells not attaching were considered PMCs. Non-separated cells (all) were cultivated in non-coated plates.

### Transfection with siRNA

Silencer Select siRNAs (S32107 and S32108 for *LRRC8A* or negative control No. 2 ID: 4390846) were obtained from Thermo Fisher Scientific and 0.57 × 10^5^/cm^2^ cells were seeded and transfected the next day with 2.5 pmol siRNA and 0.5 μl Lipofectamine RNAiMAX (Thermo Fisher Scientific) in OptiMEM I (Thermo Fisher) according to manufacturer’s instructions.

### Proliferation assay

For proliferation analysis, 2.1 × 10^4^/cm^2^ cells were seeded. After 24 hours of seeding, cells were transfected as described above. After 8 hours of transfection and every 24 hours cells were detached and counted with Fluidlab R-300 (Anjavo) cell counter device. A growth curve was generated and an exponential regression (formula: N_t_ = N_0_ × e^λt^) was calculated. Growth factor λ was used to calculate doubling times t_d_ (formula: t_d_ = ln(2) / λ).

### Membrane potential measurement

For membrane potential measurements, 1.5 × 10^4^ cells were seeded per well in a 96-well plate (9 wells per condition). If indicated, cells were transfected 24 hours after seeding with siRNA as described above. Cells were stimulated with 2 mM CaCl_2_ 48 or 24 hours before measurement and the earliest 6 hours post-transfection. To prepare the measurement, cells were stained in imaging buffer (144 mM NaCl, 5 mM KCl, 2 mM CaCl_2_, 1 mM MgCl_2_, 10 mM HEPES, and 10 mM Glucose, pH 7.4) with 1 μM DiBAC_4_(3) (Biotium) for 60 minutes at 37 °C. Fluorescence was measured using the Infinite M200 microplate reader (Tecan Trading AG) with an excitation wavelength of 488 ± 9 nm and emission at 530 ± 20 nm. If indicated the buffer was replaced by imaging buffer with 1 μM DiBAC_4_(3) and 20 μg/ml gramicidin (Sigma Aldrich) and incubated for another 30 minutes before the fluorescence measurement was repeated. The mean of technical replicates was normalized to the mean of ctrl/siCtrl at 0 hours.

### RT-qPCR

Total RNA was isolated using the NucleoSpin RNA isolation kit (Macherey&Nagel), and transcribed with the High-Capacity RNA-to-cDNA Kit (Thermo Fisher Scientific). cDNA was subjected to RT-qPCR using predesigned TaqMan Gene Expression Assayprobes (Thermo Fisher Scientific) on Step One Plus PCR System (Applied Biosystem). mRNA expression was normalized to *RPLP0* and relative changes in the respective mRNA were quantified by the 2^−ΔΔCt^ method.

### Transcriptome data analysis

RNA was isolated using NucleoSpin RNA isolation kit (Macherey&Nagel). The TruSeq RNA Library Prep Kit version 2 from Illumina was used with an input of 500 ng total RNA to prepare the library. Reads were trimmed with Trimmomatic 0.39 (Bolger et al, 2014), aligned to the hg19 reference genome using Hisat2 (2.2.1) (Kim et al, 2019). Output files were sorted using SAMtools (1.10) and read and counted using HTSeq (1.99.2). Counts were normalized using DESeq2 (1.32.0) (Love et al, 2014).

### Western blot analysis

Cells were lysed in RIPA lysis buffer (Cell Signaling Technology). Protein amounts were normalized, subjected to SDS–PAGE, and blotted onto PVDF membranes. After blocking in 5% milk/TBS-T, membranes were probed with the indicated antibodies and visualized with HRP-conjugated secondary antibodies using SuperSignal West Pico PLUS Chemiluminescent Substrate (Thermo Fisher Scientific).

### Immunohistochemistry and immunofluorescence staining

Five healthy volunteers and 10 patients with psoriasis gave written informed consent for skin biopsies. The study was approved by the ethics committee of the Clinic of the Goethe-University (116/11); written, informed consent was obtained from all patients and control participants. The Declaration of Helsinki protocols were followed. Punch biopsies (6 mm) were taken, fixed in 4% paraformaldehyde and paraffin-embedded. 4 μm sections were prepared and deparaffinized. For immunohistochemistry, the primary anti-LRRC8A antibody (NBP2-32158 1:100, Novus Biologicals) or concentration-adjusted isotype control antibody (Cell Signaling Technology) was applied overnight after antigen retrieval with EDTA solution. Histofine Simple Stain AP Multi (Medac Diagnostika) was used for detection, according to the manufacturer’s instructions. Nuclei were stained with hematoxylin. For immunofluorescence staining, specimens were rehydrated and stained with primary anti-LRRC8A antibody (NBP2-32158 1:100, Novus Biologicals) and anti-NGFR (MA5-13314 1:50, Thermo Fisher Scientific) or concentration adjusted isotype control antibody (Cell Signaling Technology) overnight, followed by staining with AlexaFluor488 and AlexaFluor594 labeled secondary antibody (1:1000, Thermo Fisher Scientific). Nuclei were stained with DAPI. Images were acquired by using a Nikon Eclipse Ci microscope. Staining intensities were evaluated by 2 independent scientists on a scale from 0 to 3 and the means were calculated for each sample.

### Statistical analysis

Statistical analysis was performed using GraphPad Prism version 8.4.3 as indicated. *P* values < .05 were considered statistically significant and were indicated as follows: ∗: *P* < .05; ∗∗: *P* < .01; ∗∗∗: *P* < .001; ∗∗∗∗: *P* < .0001.

## Data Availability Statement

The RNAseq data discussed in this publication have been deposited in NCBI's Gene Expression Omnibus (Edgar et al, 2002) and are accessible through GEO Series accession number GSE289090 (https://www.ncbi.nlm.nih.gov/geo/query/acc.cgi?acc=GSE289090)

## Ethics Statement

The study was approved by the ethics committee of the Clinic of the Goethe-University (116/11); written, informed consent was obtained from all patients and control participants. The Declaration of Helsinki protocols were followed.

## ORCIDs

Magdalena Jahn: http://orcid.org/0000-0002-3053-4017

Victoria Lang: http://orcid.org/0000-0002-4152-9002

Oliver Rauh: http://orcid.org/0000-0003-1082-8656

Torsten Fauth: http://orcid.org/0000-0002-3544-6171

Claudia Buerger: http://orcid.org/0000-0002-7838-197X

## Conflict of Interest

At the time of the initial submission, TF was employed at BRAIN Biotech AG and is now an employee of Akribion Therapeutics GmbH. This change in affiliation during the revision process had no influence on the results presented in this work. TF and CB are inventors listed on the patent application PCT/EP2019/053820, which is no longer being pursued. The remaining authors state no conflict of interest.
